# Gilthead sea bream gut bacteriome as a valuable tool for seafood provenance analysis

**DOI:** 10.1128/aem.01508-25

**Published:** 2025-10-17

**Authors:** Eduardo Feijão, Irina A. Duarte, Marcelo Pereira, Pedro Pascoal, Mónica Nunes, Susanne E. Tanner, Ricardo Dias, Bernardo Duarte, Ana Rita Matos, Andreia Figueiredo, Vanessa F. Fonseca

**Affiliations:** 1 MARE - Marine and Environmental Sciences Centre & ARNET – Aquatic Research Network Associated Laboratory, Faculty of Sciences of the University of Lisbon 426093, Lisbon, Portugal; 2 Department of Plant Biology, BioISI— BioSystems & Integrative Sciences Institute, Faculty of Sciences of the University of Lisbon 426093, Lisbon, Portugal; 3 Department of Animal Biology of the Faculty of Sciences of the University of Lisbon, Lisbon, Portugal; 4 Department of Plant Biology of the Faculty of Sciences of the University of Lisbon, Lisbon, Portugal; 5 cE3c—Center for Ecology, Evolution and Environmental Changes & CHANGE—Global Change and Sustainability Institute, Faculty of Sciences of the University of Lisbon 426093, Lisbon, Portugal; Norwegian University of Life Sciences, Ås, Norway

**Keywords:** biomarkers, fish provenance, gut bacteriome, metagenomic, *Sparus aurata*

## Abstract

The increasing demand for high-quality seafood underscores the significant challenges posed by rampant seafood fraud. This study aimed to identify regional capture biomarkers by using the gut bacteriome of *Sparus aurata* specimens through state-of-the-art long-read sequencing allied to machine learning tools. The gut bacteriomes of animals from four different fishing areas on the Portuguese coast were sequenced. The alpha and beta diversity analyses were shown to enable Center-South gut bacteriome differentiation from other fishing areas due to higher abundance of species of the phyla Pseudomonadota, Bacteroidota, and Bacillota and classes such as Alphaproteobacteria, Betaproteobacteria, and Bacilli. The gradient boosting machine (GBM) model selected by the H2O automatic machine learning pipeline presented a high global accuracy during training and validation phases, identifying Center-South and South sample provenance with 100% and 71.1% accuracy, respectively. By integrating the most important OTUs to the GBM model with the regional biomarkers identified through point biserial correlation analysis (*indicspecies* packages), a reduced set of five provenance biomarkers was identified, belonging to Gammaproteobacteria, Betaproteobacteria, and Bacilli classes, possibly highlighting the anthropogenic activities surrounding the fishing areas and local environmental abiotic factors. This study highlights the extensive and valuable information obtained by long-read sequencing and couples it with the potential of machine learning algorithms to ultimately demonstrate its efficiency in providing efficient and highly accurate seafood provenance biomarkers. This study also reports the likely influence of industrial and recreational activities, population density, and water management facilities on the gut bacteriome of *S. aurata*.

**IMPORTANCE:**

This study significantly contributes to a topic of utmost importance—seafood provenance analysis and seafood fraud—by leveraging gut bacteriome profiling. Through the application of long-read sequencing and machine learning, it identifies reliable biomarkers that distinguish gilthead sea bream from different fishing areas. These findings enhance traceability methods by providing a robust tool to combat seafood fraud and ensure food authenticity, thereby protecting the supply chain, the consumer, and the environment. Additionally, this study explores the possible interactions between the gut bacteriome and the industrial, recreational, and environmental factors that could influence the identified biomarkers of regional provenance while also offering insights into the composition of the marine ecosystems surrounding the fishing areas. This approach has broader implications for fishery management, sustainable sourcing, and regulatory enforcement.

## INTRODUCTION

According to the Food and Agriculture Organization (FAO), seafood-derived proteins account for 17% of global protein consumption (1). The increasing demand for high-quality seafood availability by an ever-growing human population has brought to the attention of stakeholders, consumers, producers, fishers, and managers the challenges induced by widespread seafood fraud activity and unsustainable fishing practices. Naturally, with consumption rates and revenue increasing, so does the potential for food fraud motivation, which involves misleading the consumer for financial gain and poses a risk to food integrity, the economy, and public health (2). The seafood supply chain is vulnerable to various types of fraud related to adulteration, provenance, and ethical trade of seafood products, including catch method fraud, illegal, unreported, and unregulated (IUU) fishing practices, and species substitution (3, 4).

To overcome these issues, an array of different practices and countermeasures from multiple stakeholders, including private label owners, international institutions, and lawmakers, have been implemented throughout the supply chain to detect and impair malpractices (3, 5). Nonetheless, biochemical and high-throughput screening techniques, including elemental fingerprinting (6–8), fatty acid analysis (9, 10), stable isotope analysis, and DNA profiling (11), have already been applied with promising results in the field of food traceability.

As an emerging tool for evaluating fish provenance, the gut bacteriome has been the subject of studies to evaluate the complex relationships between microorganisms and their hosts (12, 13). Early studies revealed the significant influence of microbial communities on the digestive and immune functions of host organisms as well as their susceptibility to changes influenced by various host-related factors (14, 15). Notably, researchers have found that habitat and host selection play crucial roles in shaping the composition of gut bacteria in fish (14, 15). In recent decades, long-read Oxford Nanopore sequencing has been rapidly evolving, bypassing the pitfalls that involve high error rates, flow cell compatibility, and cost management, garnering attention owing to its potential, reliability, and on-site use (16–18), making it a tempting high-throughput sequencing tool to analyze the genetic diversity and ecosystem structure (19, 20). This potential has also recently been explored for the detection and characterization of various biomarkers of seafood authenticity and provenance (19, 21, 22).

*Sparus aurata*, commonly known as the gilthead sea bream, belongs to the Sparidae family and Perciformes order and can be found in the warm and euryhaline waters of the Mediterranean Sea and North-East Atlantic Ocean (23). *S. aurata* is a highly valuable species, considering both capture fisheries and large-scale aquaculture, with considerable consumer value attributed to its excellent flesh quality and availability (rapid growth). Global catches of Gilthead sea bream were estimated at 13.9 tons in 2021, with the EU providing over 27% of these catches, from countries including France, Greece, Spain, Italy, and Portugal (24).

In Portugal, a country that consistently appears in the top tier globally for fish consumption per capita, the capture of wild *Sparus aurata* specimens amounted to 219 tons in 2022. Notably, the price of these specimens increased by 8.7% compared with that in the previous year. As of 2022, *S. aurata* specimens were sold at an approximate price of 13.74 €/kg (25). Owing to the increasing importance of commercial and recreational fishing, and the consequent high value of wild-caught specimens, cases of fraud, especially those related to the catch method, have been reported (26).

Considering the pitfalls and challenges within the current fishing industry, and with the goal of fostering controlled, regulated, and environmentally conscious commerce, this study aimed to identify robust biomarkers for ascertaining the geographical origin of wild-caught sea bream along the coast of Portugal by applying long-read sequencing of the gut metagenome together with machine learning models.

## MATERIALS AND METHODS

### Study areas and sample collection

**Fig 1:**
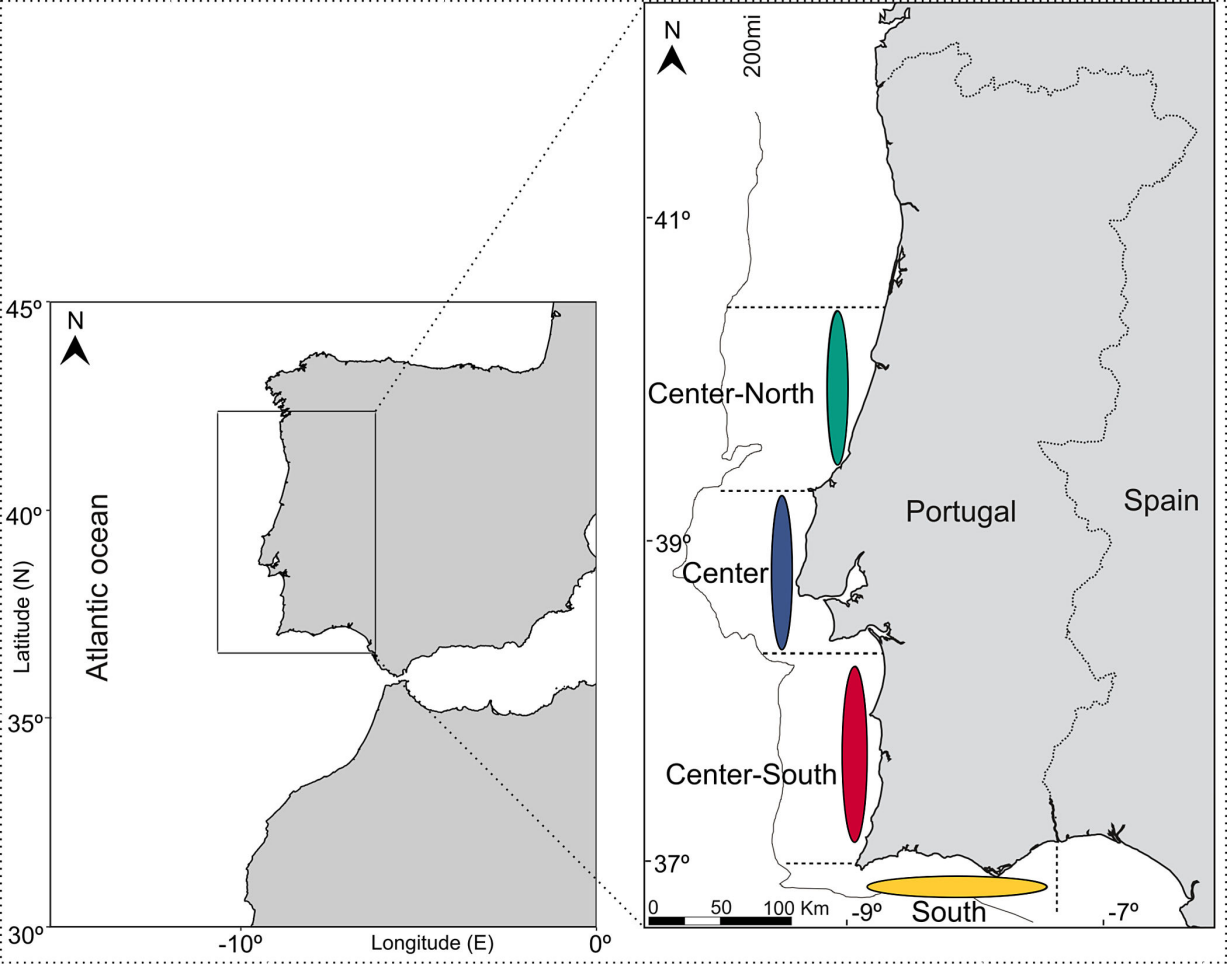
Location of the four sampling areas along the Portuguese west and south coasts. Center-North sampling area (green); Center sampling area (blue); Center-South sampling area (red); South sampling area (yellow). Map generated using coastline data from the Global Self-consistent, Hierarchical, High-resolution Shoreline Database (GSHHS).

*Sparus aurata* samples were collected in 2020 at four locations ranging from the upper geographical limit of its distribution at the Center-North (Peniche) of Portugal to the Center (Sesimbra), Center-South (Sines), and including the South of Portugal (Olhão), encompassing a fine-scale spatial distribution of approximately 500 km of marine coastal waters (Fig. 1). Maps were generated in R-Studio (R version 4.3.2 – “Eye Holes”) using coastline data from the Global Self-consistent, Hierarchical, High-resolution Shoreline Database (GSHHS, 27).

Thirty specimens with similar size and weight range (mean length = 331.78 mm ±21.73 mm; mean weight = 538.39 g ±104.71 g) were collected in each area, transported immediately to the laboratory, and post-mortem dissected to sample the intestinal tract, specifically 1 cm of intestine just above the anus, and stored at −80°C until further analysis. For morphological parameters, see Table S1.

### DNA extraction and amplification

Total DNA extraction was performed using the MGIEasy Nucleic Acid Extraction kit (MGI Tech, USA), according to the manufacturer’s instructions using the MGISP-960 robot. Genetic material quality and quantity were assessed using the NanoDrop One and Qubit 4 Fluorometer analysis. For the rDNA gene amplification, Long Amp hot start Taq 2 × master mix (New England Biolabs, MA, USA) was used at 1 x along with 50 ng/µL of genomic DNA. To amplify the full-length 16S rDNA bacterial gene, 0.25 µM of the primer pair 27F (5′-AGAGTTTGATCMTGGCTCAG-3′) and 2R (5′-CGGTTACCTTGTTACGACTT-3′) was used. PCR was conducted on a Biometra UNO II, using the following conditions: 1 cycle of 94°C for 1  min, 35 cycles of 94°C for 20 s, 55°C for 30 s, and 65°C for 2 min and a final extension of 65°C for 5 min. Subsequently, amplification products were visualized through gel electrophoresis and purified using the Solid Phase Reversible Immobilization (SPRI) technique with magnetic beads (28, 29). Quantification steps were performed using the 1x dsDNA HS assay for Qubit.

### Metagenomic sequencing by long-read Nanopore quantification

DNA was end-repaired with NEBNext End Repair Module (New England BioLabs, MA, USA), cleaned with Agencourt AMPure XP Beads (Beckman Coulter, High Wycombe, UK), and dA-tailed (New 160 England BioLabs, MA, USA). The library was prepared from 300  ng input DNA from each sample using the Sequencing Native Barcoding Kit 24 V14 (SQK-NBD114.24) (Oxford 162 Nanopore Technologies, Oxford, UK) in accordance with the manufacturer’s protocol. The library was quantified and prepared for PromethION sequencing, using FLO-PRO114M flowcells, MinKNOW v22.12.4, and standard 72 h run script with active channel selection enabled. DNA flow rate was 400 bases/s. After 24 h, 3,000,000 passed reads were yielded with an estimated N50 of 1,500 bp, and the mean quality score was 14. In total, 4.5 Gb of data was produced, with an average of 750,000 reads per sample. Data were collected using the Epi2Me software (Oxford Nanopore Technologies, UK).

### Bioinformatic analysis

Following the removal of low-quality gDNA reads, the remaining reads were filtered using Prinseq-lite version 0.20.4, keeping reads with length and Phred score higher than 300 bps and 7, respectively (30). Taxonomical classification was performed using an in-house analytical pipeline for long-read Nanopore sequencing, as described by (31). Taxonomic classification was obtained through Kraken2 (version 2.1.2) with default settings and the NCBI Refseq database (Bacteria) to merge assignments into the highest common taxonomic rank (LCA) (31). Each taxonomic rank classification was considered an operational taxonomic unit (OTU), allowing for a direct interpretation of taxonomic diversity without the need for traditional clustering methods.

The following analyses were performed using the R-Studio version 4.3.2 – “Eye Holes.” Only the OTUs present in more than 25% of the samples were included in the alpha and beta diversity analyses.

Rarefaction, alpha, and beta diversity analyses were performed using the *phyloseq* package (32). Statistical analysis of the different diversity parameters and data abundances across all sampling sites was conducted using the Kruskal-Wallis tests, with the *ggpubr* package used for improved data visualization (33). Further comparative analysis of taxa abundance was performed using phylogenetic heat trees and the *metacoder* package (34). To limit potential taxonomic bias at higher taxonomic levels, only OTUs with a complete taxonomic lineage were used, and standard data preprocessing procedures following package guidelines were implemented. OTUs with zero and low abundance (counts < 5) were excluded, and data normalization for uneven sampling was performed following the method proposed by (34). Visualization was accomplished using the Davidson-Harel layout, with node placement facilitated by the Reingold-Tilford algorithm. Further analysis involved evaluation of significant differences in taxonomic abundance between sample groups using the built-in Wilcoxon test within the *metacoder* package.

For the traceability model development, the H2O Automatic Machine Learning pipeline was used (35, 36). The training and prediction phases were conducted after randomly partitioning the data set into training (70%) and testing (30%) subsets. The tested models included deep learning, distributed random forests (DRF), generalized linear models (GLM), extreme gradient boosting (XGBoost), gradient boosting machines (GBM), and stacking ensemble. A maximum of 20 models were generated, and their performance was evaluated using the H2O Automatic Machine Learning pipeline. The generated models were evaluated based on their test and training accuracy. The GBM model was selected using the following parameters. Fivefold cross-validation used, and the score of the model was evaluated after every five trees. A total number of 40 trees was used with a maximum tree depth of 8. Feature importance was calculated using the H2O automatic machine-learning pipeline for the best-performing model.

The indicator OTUs for each fishing area gut bacteriome were extracted using point biserial correlation analysis using the *indicspecies* packages (37, 38) and filtered according to the most important OTUs in the prediction model. The most important OTUs in the model were those that cumulatively explained 80% of the results. Biomarker abundance within the provenance categories and accumulative abundance were assessed using the *agricolae* package, and statistical analysis was performed using the Kruskal-Wallis analysis (39).

## RESULTS

### Long-read sequencing analysis

The gut bacteriome of *S. aurata* specimens was sequenced using long-read Oxford Nanopore sequencing technology. In total, 187 thousand reads were registered, 180 thousand of which were used for taxonomic construction using a custom in-house pipeline that divided the obtained data into sets of sequences of length ~1,300 bp. As a result of the first data processing, it was possible to identify 47,390 OTUs that belonged to 92 phyla, 91 classes, 225 orders, 586 families, 2,935 genera, and 38,607 identified species. After further processing, which included discarding OTUs that were not present in at least 25% of the samples, 1,804 OTUs that belonged to 5 kingdoms, 23 phyla, 40 classes, 90 orders, 190 families, 446 genera, and 1,577 species were used to analyze alpha and beta diversities.

### Alpha and beta diversity analysis

**Fig 2:**
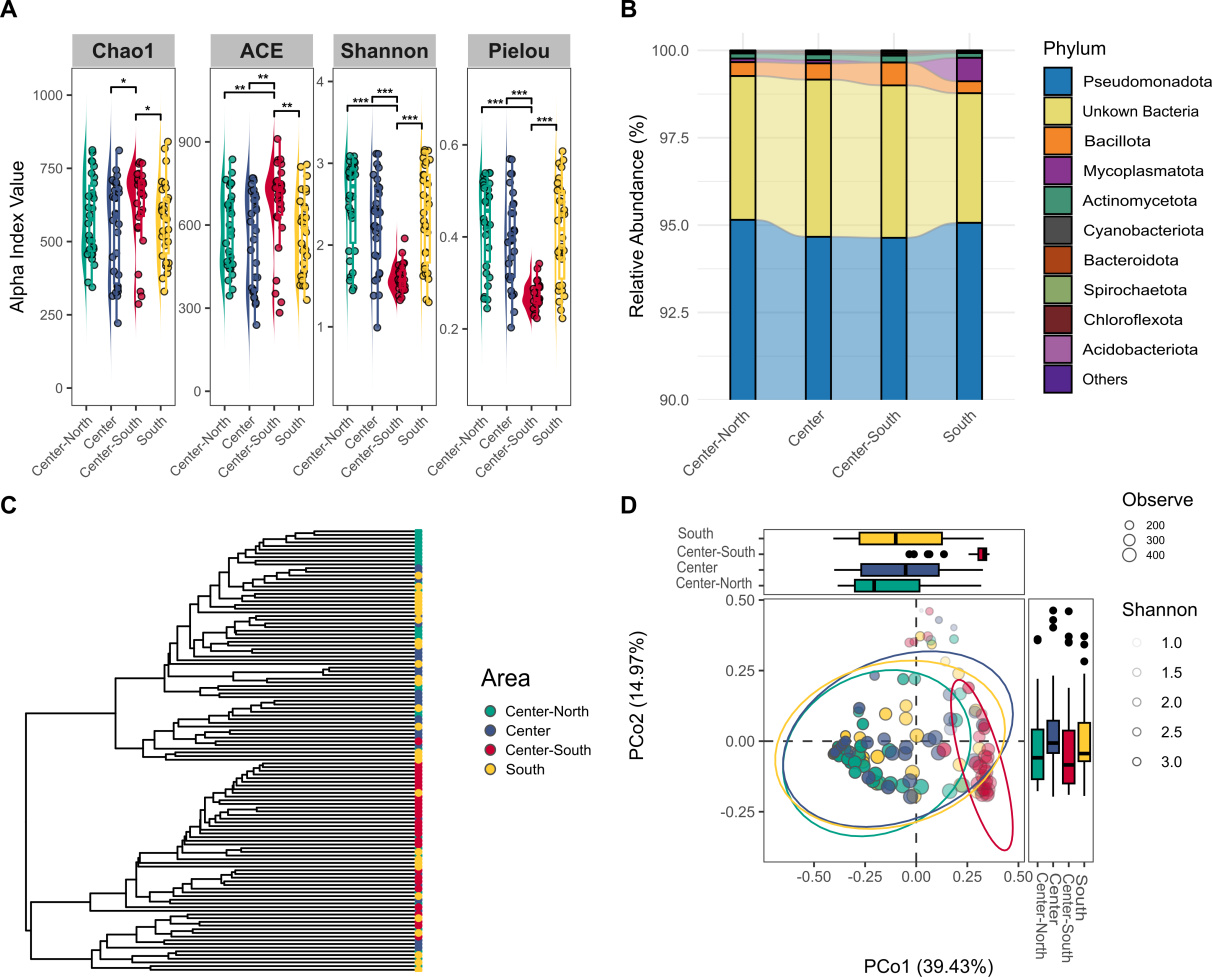
Diversity analysis of *S. aurata* gut bacteriome samples from four fishing areas along the coast of Portugal. (**A**) Alpha diversity analysis; (**B**) relative abundance (%) of the top 10 most abundant phyla; (**C**) cluster analysis based on Jaccard dissimilarities; (**D**) principal coordinate analysis (PCoA); *N* = 30 replicates per site. Asterisks denote significant differences at *P* < 0.05 (*), *P* < 0.01 (**), and *P* < 0.001 (***) using the Kruskal-Wallis test.

When comparing the gut bacteriome species richness in S. *aurata* samples from different fishing areas along the coast of Portugal, as represented by the Chao1 index (Fig. 2A), the Center-South samples tended to have a significantly higher number of species when compared to other areas, excluding the Center-North fishing area. Using the abundance-based coverage estimator index (ACE) to assess OTU abundance, a similar pattern was observed in *Sparus aurata* samples from the Center-South region, which differed significantly from all other fishing areas. In contrast, no significant variation in species richness estimators was detected among the remaining fishing areas. When taking into consideration Shannon’s diversity index (H), which indicates how difficult it is to correctly predict the OTUs of a sample, significantly higher H values were observed in samples from the Center-North, followed by samples from the South and Center areas, revealing no statistical significance among them. The gut microbiome of the Center-South samples shows less diversity when compared to the gut microbiomes of the specimens prevenient of the other fishing areas (*P* < 0.01).

Considering Pielou’s evenness index, when compared to the gut bacteriome of specimens from the Center-South fishing area, samples from the Center presented significantly higher evenness, followed by samples from the Center-North and South, and Center (*P* < 0.05). However, no statistical difference was observed in evenness among the Center-North, Center, and South gut bacteriomes. Overall, the alpha-diversity analysis highlighted the Center-South as the region where the gut bacteriome of *S. aurata* samples presented higher species richness but the lowest evenness and diversity.

The most abundant phylum was Pseudomonadota, accounting for more than 90% of the population in each sampling group, followed by Bacillota, Mycoplasmatota, and Actinomycetota (Fig. 2B). Center and Center-South presented a lower relative abundance of Pseudomonadota but higher abundance of Bacillota and Actinomycetota, while the specimens from the South of Portugal presented a higher relative abundance of Mycroplasmatota.

The β-diversity analysis was evaluated through cluster analysis using the Jaccard dissimilarity matrix and PCoA, and results indicated a separation between *S. aurata* samples from the Center-North and Center-South of Portugal with overall variability in samples from the South (Fig. 2C and D). PCoA explained data variance (54.4%) in the first two components, with the first axis (PCo1) explaining the highest variance proportion (39.43%) to the overall separation of Center-South samples from most of the other samples.

**Fig 3:**
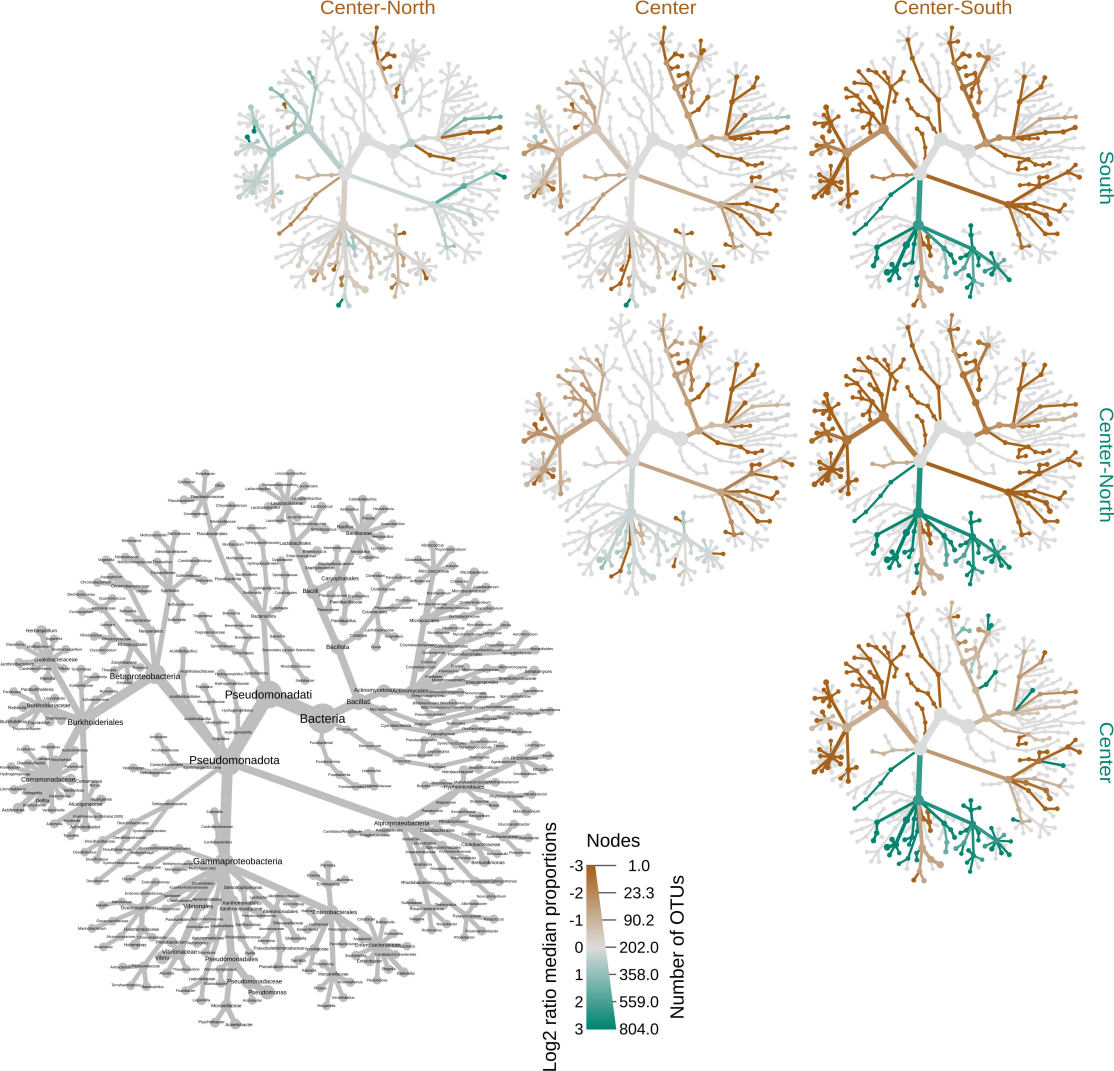
Comparative phylogenetic heat trees of the gut bacteriome of *S. aurata* samples from four different fishing areas along the coast of Portugal. *N* = 30 replicates per site; colored branches denote significantly different abundances of a specific taxon at *P* < 0.05, as determined by the Wilcoxon test.

Analysis of taxa abundance through comparative phylogenetic trees (Fig. 3) revealed differences in branch abundance across phylogenetic trees derived from samples originating from the four fishing areas. *Sparus aurata* samples from the Center-South region exhibited a significantly higher abundance of OTUs from Pseudomonadota, Bacteroidota, and Bacillota phyla when compared to other regions, which was also evident from Fig. 2B. Among the most abundant classes from these phyla present in the Center-South samples, when compared to the other fishing areas, are Alphaproteobacteria, Betaproteobacteria, and Bacilli, with a higher abundance of OTUs from orders such as Rhodocyclales, Burkholderiales, and Spirillales. It is evident that the gut bacteriome of the *S. aurata* specimens from the Center-North, Center, and South fishing areas exhibits a higher abundance of OTUs belonging to the phylum Pseudomonadota when compared to the specimens from the Center-South region. Notably, the most substantial differences are found at the class and order levels, with great abundance of OTUs belonging to classes such as Gammaproteobacteria and orders such as Enterobacterales, Vibrionales, and Oceanospirillales. Among these central regions, the abundance of OTUs related to the phyla Bacillota, Actinomycetota, Bacteroidota, and Spirochaetota seems to be significantly and progressively increasing from the Center-North to Center-South areas.

### Pattern recognition and data modeling

**Fig 4:**
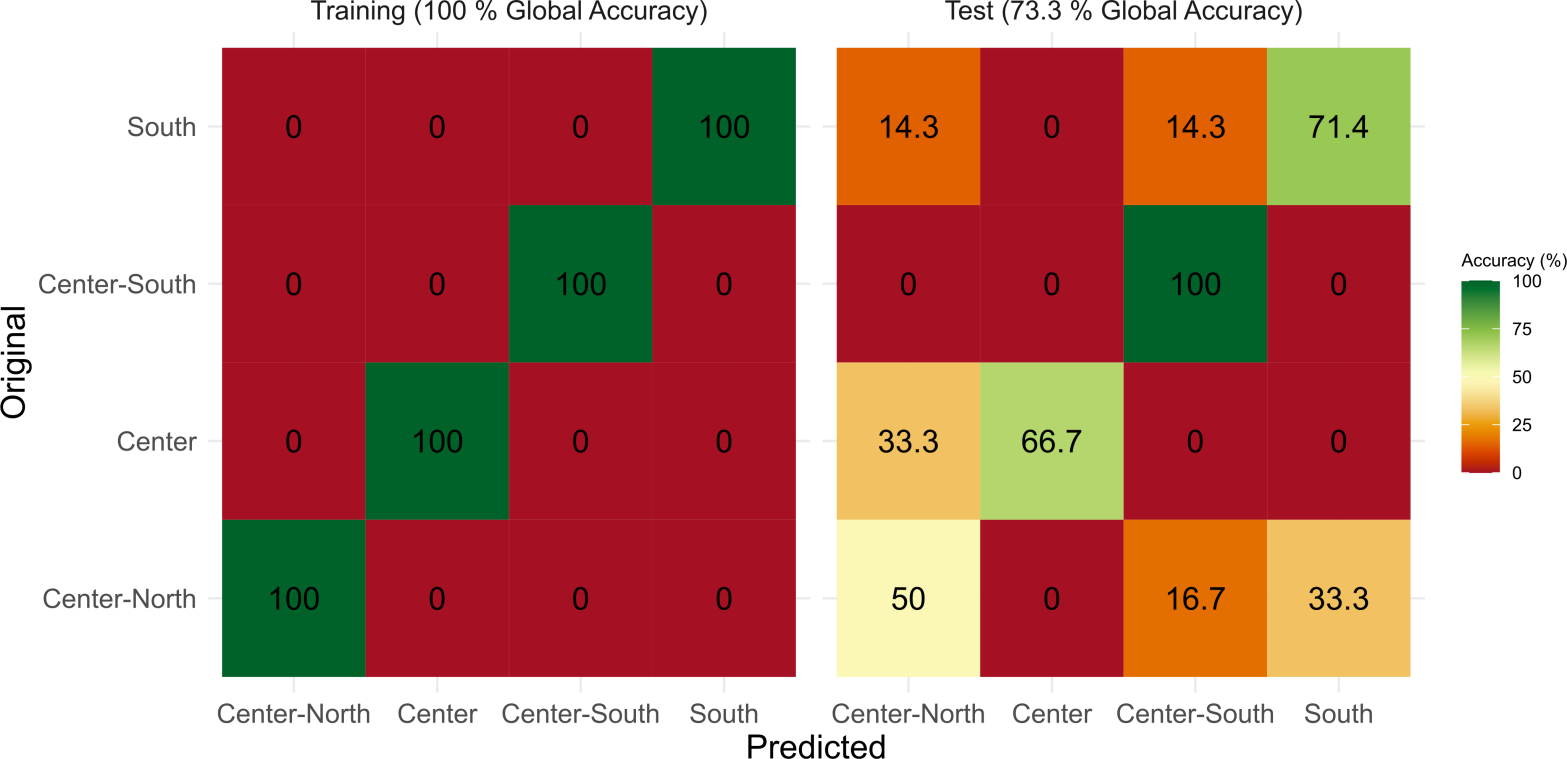
Gradient boosting machine (GBM) model training and testing accuracy heatmaps of the gut bacteriome of *Sparus aurata* samples from four different fishing areas along the coast of Portugal.

Among all the models tested, the gradient boosting machine (GBM) model achieved the best results. The results obtained from the training phase of the GBM model present in Fig. 4 (left panel) showed a 100% score of training accuracy. When using a blind test data set to test the trained model, the global accuracy of the obtained model for assigning samples to the provenance area of each test sample was still high (73.3%) (Fig. 4, right panel).

The model enabled 100% accuracy in correctly classifying samples from the Center-South area, followed by 71.4% accuracy in the classification of samples from the South fishing zone. Lower efficiencies when applying the test data set were observed in samples from the Center (66.7%) and Center-North (50%) areas, with some misclassification with the assignment of samples to the Center-North area in the first case, and in the Center-South and South fishing areas in the latter. Most misclassification errors result from sample mismatches in adjacent regions (for example, between Center and Center-North areas and between South and Center-South areas).

### Biomarker identification

To obtain the best possible biomarkers for identifying fish provenance, a chained variable selection based on two statistical methodologies was employed: the point biserial indicator species model (Indicspecies), which identifies OTUs sensitive to changes according to the grouping variable, and GBM Feature Importance, which allows the identification of the most influential variables in the abovementioned classification model.

Fish gut bacteriome indicator species analysis resulted in a selected number of exclusive OTUs in each sampling area. This analysis provided the highest number of possible biomarker candidates for *S. aurata* provenance from the Center-South (444 biomarker candidates) and Center (138 biomarker candidates) areas, with only a few biomarker candidates from the Center-North (five biomarker candidates) and South (17 biomarker candidates) fishing areas (Fig. S1, in the supplement).

**Fig 5:**
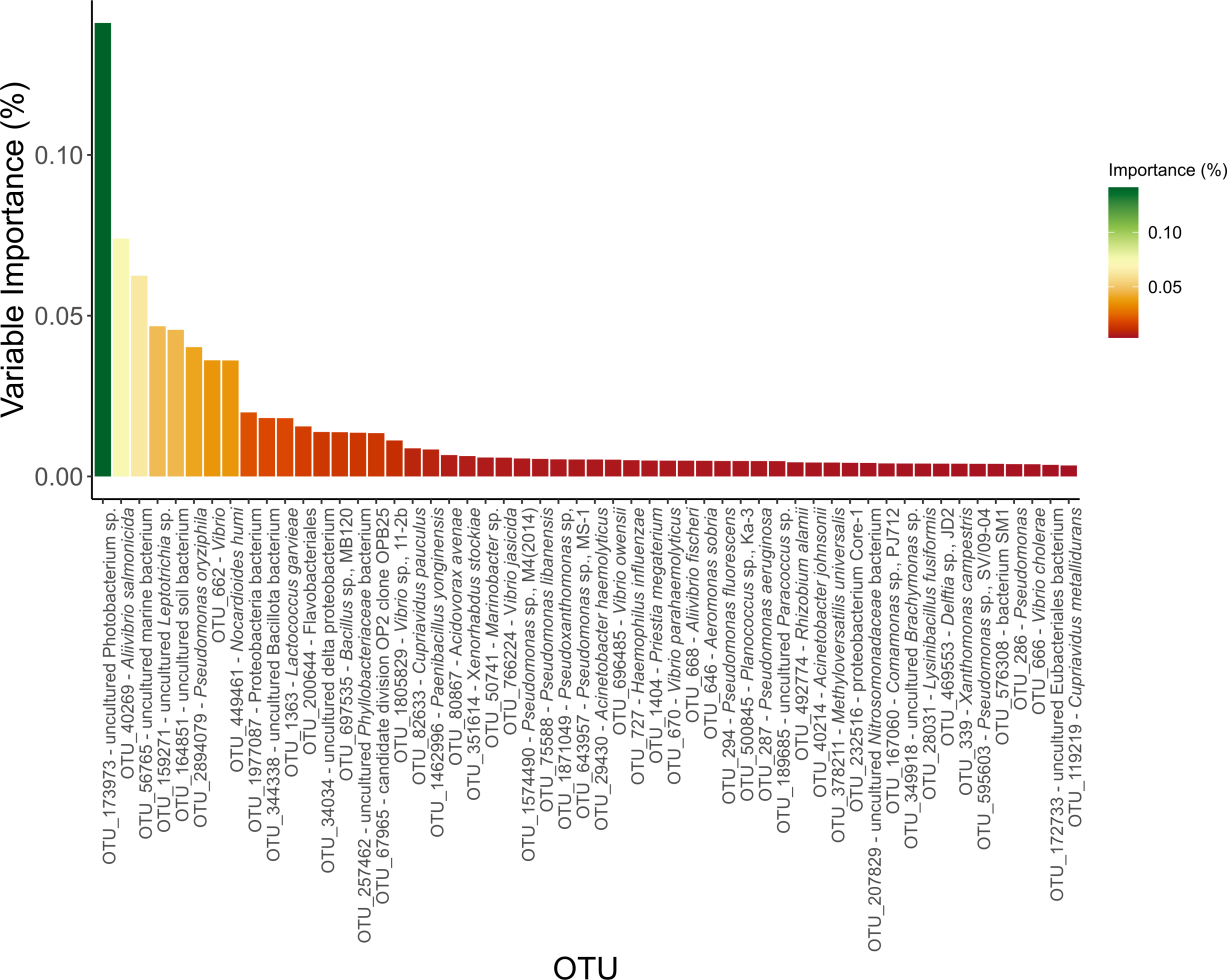
Feature importance of the most important OTUs for the prediction model (cumulative importance = 80%).

The most relevant OTUs were selected until 80% cumulative feature importance was achieved, resulting in the selection of 54 candidate OTUs with higher explanatory power, considering the traceability of the GBM model produced (Fig. 5). Subsequently, the top five biomarkers for each fishing area were obtained after merging the indicator species results with the most important OTU data for the prediction model, highlighting the common biomarkers.

**Fig 6:**
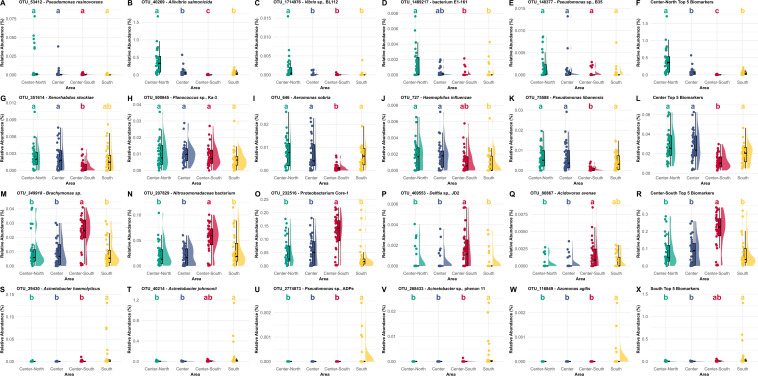
Top five proposed biomarkers of the *Sparus aurata* gut bacteriome for each fishing area to evaluate the provenance. (**A–F**) Center-North biomarkers; (**G–L**) North biomarkers; (**M–R**) Center-South biomarkers; (**S–X**) South biomarkers. Different letters denote statistically significant differences among the fishing areas obtained through a Kruskal-Wallis statistical analysis.

To assess the suitability of the proposed biomarkers individually and as part of a set of biomarkers to trace seafood provenance, the relative abundance (%) of each candidate OTU biomarker in every sample was calculated, and its abundance was compared across sampling sites (Fig. 6). A suitable biomarker will be most abundant in the region for which it is proposed as a provenance biomarker and ideally absent or with very low abundance in the gut microbiomes of the fish specimens collected in other areas.

Upon merging the two statistical approaches, it was evident that most of the OTUs identified as biomarkers of geographical origin belonged to the Gammaproteobacteria class (75%), with only a few corresponding to the Betaproteobacteria (20%) and Bacilli (5%) classes. Of all the suggested biomarkers, the Center-North area presented the only OTUs belonging to the order Vibrionales. The Center fishing area presented only OTUs from the Aeromonadales, Pasteurellales, and Caryophanales orders, while the Center-South and South regions presented only OTUs belonging to the orders Burkholderiales and Pseudomonadales, respectively.

For Center-North, this approach selected OTUs corresponding to *Pseudomonas resinovorans*, *Aliivibrio salmonicida*, *Vibrio* sp., BL112, *Bacterium* E1-161, and *Pseudomonas* sp., B35. The relative abundance of the sum of the five selected biomarker OTUs was significantly higher in the samples collected in this area than in the remaining test sites. It is important to acknowledge that the best provenance biomarkers to classify *S. aurata* samples from the Center-North are *Aliivibrio salmonicida* and *Vibrio* sp. BL112.

To evaluate the *S. aurata* provenance of the Center samples, the proposed biomarkers were *Xenorhabdus stockiae*, *Planococcus* sp., Ka-3, *Aeromonas sobria*, *Haemophilus influenzae,* and *Pseudomonas libanensis*. Upon suitability assessment, it is possible to observe that these biomarkers do not properly discriminate center-captured samples from specimens captured in the Center-North, Center-South, and South, individually and as a set of biomarkers, in line with the higher degree of misclassification observed in the GBM test set confusion matrix and from where the feature importance was extracted.

The proposed biomarkers to trace *S. aurata* gut bacteriome samples provenance to Center-South included the *Brachymonas* sp, *Nitrosomonadaceae bacterium*, *Proteobacterium* Core-1*, Delftia* sp, JD2, and *Acidovorax avenae*. These biomarkers showed statistically significant higher relative abundances in samples from the Center-South region when compared to the other areas. As a set of provenance biomarkers, these species demonstrated strong discriminatory power, both individually and collectively, effectively distinguishing between fishing areas.

To trace *S. aurata* specimens to the South fishing area, the proposed biomarkers included *Acinetobacter haemolyticus*, *Acinetobacter johnsonii*, *Pseudomonas* sp., ADPe, *Acinetobacter* sp., phenon 11, and *Azomonas agilis. Acinetobacter johnsonii* showed limited resolution, failing to distinguish between the South and Center-South regions. In contrast, the other proposed biomarkers exhibited higher relative abundance in the gut bacteriome of specimens from the South region, effectively discriminating this area from the others when analyzed individually. However, the cumulative relative abundance of the biomarkers did not show a statistically significant difference between the South and Center-South regions.

## DISCUSSION

The escalating prevalence of fish fraud poses significant risks to the environment, economy, and human health (2). This underscores the critical need to counter fraudulent activities by implementing stringent regulations. Additionally, it is of utmost importance to explore innovative techniques and determine new biomarkers to accurately determine fish identity and provenance so that we can safeguard the environment from resource exploitation. Through this approach to explore innovative techniques and determine new biomarkers, it is also possible to link high-quality fresh seafood to geographical provenance, increasing the value of current seafood products and investing in commerce, recreational fishing activities, and local seafood markets and restaurants. Ultimately, the biggest goal is to mitigate health hazards for consumers while also protecting the environment and economy. This study used long-read Nanopore sequencing to analyze the gut bacteriome of four groups of *Sparus aurata* from different fishing regions along the Portuguese coast, spanning approximately 500 km. The aim was to identify distinct biomarkers indicative of regional provenance based on their geographical distribution. The gut bacteria of fish are heavily influenced by factors such as water temperature, salinity, oxygen concentration, diet, feeding habits, habitat community, and intrinsic factors such as genetics and developmental stages (15, 40). However, it has been reported that most of the gut bacteriome of fish is composed of bacteria with a high abundance of the phyla Pseudomonadota, Bacillota, and Bacteroidota (41), with carnivorous fish showing a higher abundance of Pseudomonadota and Bacillota than herbivorous species (13, 42). While the alpha diversity analysis results of the different *S. aurata* gut bacteriome groups revealed key differences in species richness, diversity, and equitability among the gut bacteriomes, the most abundant OTUs belonged to the phyla Pseudomonadota, Bacillota, Mycoplasmatota, and Actinomycetota, which is in accordance with the aforementioned literature. When compared at the species level, our results are supported by other published studies, although the abundance of each phylum may vary according to the body part analyzed (e.g., flesh, stomach, and intestines), capture, and production methods (40, 43–45). Interestingly, comparative phylogenetic heat tree analysis revealed that gut bacteriome samples of specimens from the Center-North, Center, and South presented a higher abundance of OTUs belonging to the Gammaproteobacteria class and orders Vibrionales and Oceanospirillales, which have been reported to increase in acidic environments and are associated with various seaweed species (46). Although the algal community, surface water temperature, and ocean acidification are interactive factors in marine ecosystems, it is not surprising that these OTUs were higher in the gut bacteriome of Southern samples because of the overall higher surface water temperature that bathes the South coast with the Mediterranean Sea influence (47–51). However, it is expected that colder surface water temperatures might influence the abundance of other bacteria belonging to these orders, specifically the order Vibrionales, which includes bacteria known to thrive in cold waters (52). It is also important to note that outside of the Center-South region, *S. aurata* specimens seem to have more OTUs belonging to the order Enterobacterales, which includes cellulolytic enzyme-producing bacteria commonly found in herbivorous fish, possibly highlighting dietary differences between Center-South and the other fishing areas, as opposed to OTUs belonging to bacteria of the Bacilli class (53).

While diversity analysis was used to assess possible differences among the gut bacteriomes of *S. aurata* samples, machine learning algorithms were used to identify patterns and interactions between variables that allow extraction of fish provenance prediction models and biomarkers (54). In the training phase, the GBM model presented 100% accuracy, discriminating patterns among OTU abundance, taxonomic identification, and fishing areas. When applying the blind sample data set, the global model accuracy remained high at 73.3%, with three sites classified with accuracies above 66.7%. Notably, samples from the Center-South area presented the highest accuracy, aligned with the differences in the various diversity indices explored in the gut bacteriome. The lower accuracy of the model predictions among the other three fishing areas is also supported by the alpha diversity, cluster, and PCA analyses. These results highlight a few challenges in evaluating fish provenance, including species mobility and geographical distribution. *S. aurata* is a mobile species and therefore difficult to control its movement toward other fishing areas. The presence of this species above the Central-Northern area is not common, although it has been reported to occur more often in the Celtic Sea and in the cold waters of the English Channel (55–57).

After crossing the most important OTUs for the model with the Indicator Species analysis, it was possible to extract five biomarkers that can be used as a package tool for easier, faster, and cheaper fish food provenance analysis. Overall, the proposed biomarkers linking gut bacteriome samples to fishing areas appear to be influenced by anthropogenic activities, such as primary and extractive industries including agriculture, fishing, and mining, as well as recreational activities and environmental factors such as surface water temperature, and resistome (58–62). This approach aims to reduce the number of OTUs to be analyzed to confirm or assess the provenance of a certain *S. aurata* sample using targeted analysis while maintaining the explanatory resolution of the analyzed traits.

The Center-North region is a densely populated area, with an increased seasonal population density due to its touristic characteristics, but also includes areas of intense fishing activities, industries related to the transformation of primary products, and agricultural activities (61, 63). The high relative abundance of *Aliivibrio salmonicida*, a gram-negative bacterium and, to a lesser extent, Vibrio sp. BL112 may be related to water temperature because it thrives in cold waters (52). The Portuguese coast experiences some variation in surface water temperature; therefore, it is not surprising to find these bacteria more commonly near the northern coast and outside the warmer waters of the southern Portuguese coast (47, 62). Information regarding *bacterium* E1-161 is scarce, and its taxonomical classification places it in the order Enterobacterales. Bacteria from this order are often reported to develop resistance to antibiotics due to their ability to produce β-lactamases, β-lactam antibiotic degrading enzymes, rendering their efficacy null (64). Additionally, it is interesting to observe that the relative abundance of this specific biomarker was similar in the Center-North and Center regions, neighboring areas with high coastal population density. This is in line with previous studies, where the occurrence of antibiotic resistance genes was directly related to environments subjected to high anthropogenic pressure (58). Agricultural activities are also prominent throughout the country, so it was no surprise that biomarkers related to the *Pseudomonas* genus (*P. resinovorans* and *Pseudomonas* sp. B35), including pesticide-tolerant bacteria (e.g., *Pseudomonas resinovorans* SZMC 25872), did not show differences in relative abundance among the fishing areas (65). Although individually, these two biomarkers present lower resolution compared to the other biomarkers, to assign a certain gut microbiome sample as originating from the Center-North area, when grouped as a provenance discrimination set, the discrimination success was statistically validated.

The proposed biomarkers for tracing *S. aurata* provenance to the central region included *Aeromonas sobria* and *Haemophilus influenzae,* which have been shown to develop antibiotic resistance (66, 67), often correlated with high population densities (68). This could be related to previously reported high concentrations of antibiotics present in the waters of the Tagus estuary, which is surrounded by the Lisbon metropolitan area, an area with approximately 2.5 million inhabitants (59). The other proposed biomarkers include *Pseudomonas libanensis,* which is a denitrifying bacterium that has also been isolated from wastewater. Again, the large wastewater outfall from the Lisbon Metropolitan area may be responsible for the high abundance of this bacteria in the specimens captured in the vicinity of this large urban center (69–71); the *Xenorhabdus stockiae* bacterium could also be related to the extensive agricultural activity in this region (72). Information regarding Panococcus sp., Ka-3 is scarce, although some strains have been reported to be able to use hydrocarbons as carbon sources, such as those abundant near port areas (73). Two of the most prominent ports in Portugal (Lisbon and Setúbal) could explain the higher abundance of these bacteria in the gut microbiomes of species captured in the Center area (74). Although the resolution and discrimination power of this group of biomarkers were low, tracing *S. aurata* specimens to the Center fishing area will ultimately rely on the strength of the biomarkers from the other areas. Specimens caught in the Center can be identified by a process of exclusion when they do not match the biomarker profiles of the other areas.

The biomarkers used to trace *S. aurata* samples to the Center-South region were successfully identified, and both their resolution as an individual and a set of provenance biomarkers were remarkable. The relative abundance of the top five biomarkers was higher in samples from the Center-South than in any other fishing area, and the same pattern was observed for each biomarker. This results from the high accuracy of the GBM classification for this area (100% accuracy) and is thus reflected in the selected high-importance features. This group of biomarkers is likely linked to three major industrial and commercial activities in the study area, a highly populated and industrialized region. These activities include: (i) the commercial port of Sines, the main port in the Ibero-Atlantic front, which is the country’s leading energy supplier of crude and its derivatives (coal and natural gas); (ii) the remaining cattle breeding activities and/or intense agricultural exploitation; and (iii) the mining activity that surrounds the area that includes the Aljustrel mines (60, 75).

These activities could be sources of environmental contamination with heavy metals such as Cr, As, Pb, Cd, Cu, Fe, and Zn, as well as other contaminants such as ammonia and other nitrogen compounds (60, 76). This group of biomarkers includes heavy metal-tolerant bacteria such as the uncultured *Brachymonas* sp. and the ammonia-oxidizing *Nitrosomonadaceae* bacterium, which has been reported to be resistant to a wide array of metals and metalloids commonly found near industrial areas (As, Pb, Cd, Cu, and Zn) (77); and Pb(II) and Cr(II)-resistant *Delftia* sp. bacteria (78). The bacterium *Acidovorax avenae* has been reported to degrade chlorobenzenes, which are widely used in many industries (e.g., pharmaceuticals, agriculture, dyes, and petrochemical industries) and often reach water bodies through involuntary discharges (79–81). Finally, Proteobacterium Core-1 belongs to the Gammaproteobacteria class, and while this specific bacterium and its presence in the gut bacteriome of *S. aurata* requires more extensive research, possible cross-contamination between soil and surface water bodies cannot be excluded since this class has been known to respond to different concentrations of environmental soil pollution (82, 83).

The *Acinetobacter* genus includes three reported bacteria in the set of biomarkers presented as candidates for classifying gut bacteriome samples from specimens captured in the southern area of Portugal. This group of biomarkers has been reported to be heavy metal-tolerant and abundant in WWTPs, similar to the other OTUs mentioned above. Nevertheless, the higher water temperatures in this area of Portugal could influence their abundance (84, 85). The *Acinetobacter johnsonii* and *Acinetobacter haemolyticus* are tolerant to metals such as copper, lead, and chromium (84–86). Nonetheless, bacteria of this genus seem to prefer warmer temperatures around 25°C, which can be found in the Southern Portuguese coastal waters and match th*e* optimal growth temperatures (22°C to 26°C) of *S. aurata* (62). This abiotic factor seems to be influencing other biomarkers such as *Azomonas agilis,* which prefers warmer surface water temperatures (87).

The last proposed biomarker is the *Pseudomonas* sp. ADPe, which could be related to the high abundance of golf courses along the South region, specifically in Algarve, because of its capacity to degrade atrazine, a chlorinated herbicide used to control broadleaf weed growth (88–90). However, this genus of bacteria is often naturally present in golf courses, and one cannot exclude environmental cross-contamination (91).

Overall, long-read Oxford Nanopore sequencing provided comprehensive insights into the gut bacteriome of *Sparus aurata*, leveraging detailed OTU abundance data to identify a practical and accurate set of biomarkers for determining fish provenance. Additionally, this study highlights the impact of industrial and recreational activities, population density, and water management infrastructure on coastal ecosystems, with a particular focus on their effects on the gut bacteriome of *S. aurata*. Future work should improve on the proposed model (e.g., by increasing sample size), evaluate biomarker stability through time and seasons, test the present or improved methodology on other species and/or farming practices, and validate these biomarkers.

### Conclusion

The gut bacteriome of *S. aurata* has emerged as a valuable tool for discerning regional variation among different fishing areas. Through diversity analysis, distinct patterns in OTU abundance were uncovered in the Center-South samples, which exhibited a higher prevalence of OTUs associated with Rhodocyclales, Burkholderiales, and Spirillales orders. Conversely, other fishing areas displayed an elevated abundance of OTUs attributed to orders Enterobacterales, Vibrionales, and Oceanospirillales. These differences were also evident in the top five biomarkers in each fishing area.

Employing machine learning techniques yielded remarkable efficiency during the training phase, achieving a perfect accuracy score. Furthermore, these methods successfully identified the origin of the gut bacteriome from blind samples with a commendable accuracy of 73.3%, notably by linking the provenance of blind and random *S. aurata* gut bacteriome samples to the Center-South.

Building on these findings, this study proposed 20 potential biomarkers for evaluating the provenance of *Sparus aurata*. These biomarkers offer promising avenues for enhancing traceability efforts within the fishery industry, facilitating more precise regional assessments, and bolstering quality control measures along the supply chain.

The proposed biomarkers for tracing *S. aurata* provenance appear to reflect regional environmental pressures, including seasonal human activity, water temperature gradients (e.g., *Aliivibrio salmonicida* and *Vibrio* sp., BL112), and agriculture (e.g., *Pseudomonas resinovorans* and *Pseudomonas* sp., B35) in the Center-North; urbanization (e.g., *Aeromonas sobria* and *Haemophilus influenzae*), wastewater discharge (e.g., *Pseudomonas libanensis*), agricultural runoff (e.g., *Xenorhabdus stockiae*), and port activity (e.g., *Panococcus* sp., Ka-3) in the Center; industrial and port activity pollution, adjacent mining activities, and heavy metal exposure (e.g., *Brachymonas* sp., *Nitrosomonadaceae*, and *Delftia* sp.) in the Center-South; and warmer water temperatures (e.g., *Azomonas agilis*), WWTP density (e.g., *Acinetobacter johnsonii* and *A. haemolyticus*), and recreational land use (e.g., golf courses; *Pseudomonas* sp., ADPe) in the South.

## Data Availability

Raw data were submitted to the National Center for Biotechnology Information (NCBI) under the Sequence Read Archive (SRA) accession no. PRJNA1330551.

## SUPPLEMENTAL MATERIAL

Supplemental material aem.01508-25-s0001.docx Table 1, *S. aurata* morphological parameters; Fig. S1, indicator species results.

ASM does not own the copyrights to Supplemental Material that may be linked to, or accessed through, an article. The authors have granted ASM a non-exclusive, world-wide license to publish the Supplemental Material files. Please contact the corresponding author directly for reuse.
