# Supplementary material for: Gilthead sea bream gut bacteriome as a valuable tool for seafood provenance analysis
Source: Appl Environ Microbiol. 2025 Oct 17;91(11):e01508-25. doi: 10.1128/aem.01508-25 (PMC12628811; doi:10.1128/aem.01508-25)
Supplement: Supplemental material — Table 1, S. aurata morphological parameters; Fig. S1, indicator species results. [file aem.01508-25-s0001.docx]

**Supplementary Table S1** – Morphological parameters of S. aurata specimens. The values correspond to average and standard errors. N = 30 samples.

| **Area** | **Length (mm) ± SE** | **Weight (g) ± SE** |
| --- | --- | --- |
| Center-North | 315.33 ± 2.06 | 580.47 ± 8.66 |
| Center | 339.13 ± 2.38 | 585.75 ± 10.74 |
| Center-South | 346.60 ± 4.26 | 577.04 ± 21.89 |
| South | 324.07 ± 4.46 | 571.92 ± 19.84 |


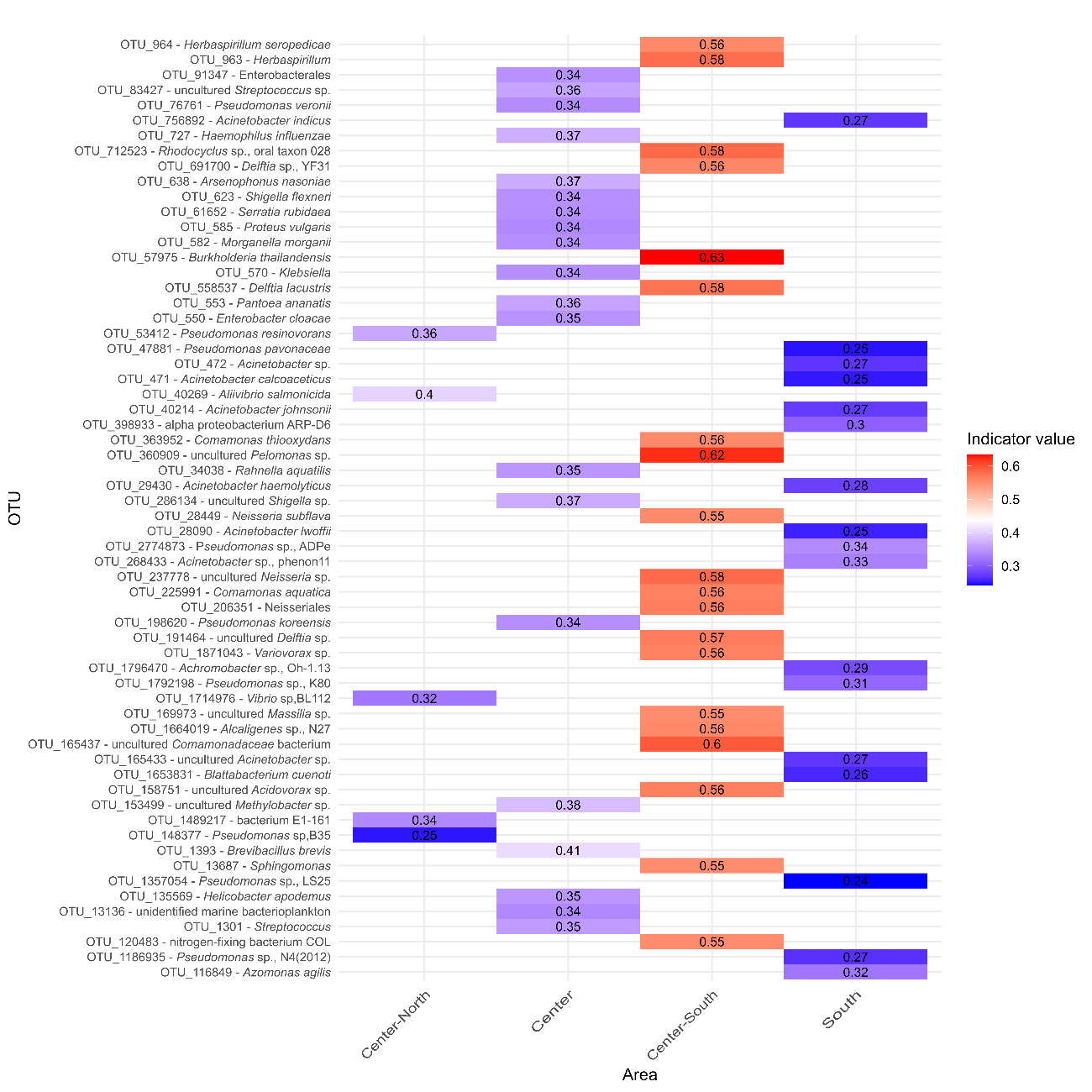


**Figure S1** – Highest biomarker indicator value per fishing area suggested by indicator species analysis with their respective indicator values.
